# The psychology of Bayesian reasoning

**DOI:** 10.3389/fpsyg.2014.01144

**Published:** 2014-10-09

**Authors:** David R. Mandel

**Affiliations:** Socio-Cognitive Systems Section, Defence Research and Development Canada and Department of Psychology, York UniversityToronto, ON, Canada

**Keywords:** Bayesian reasoning, belief revision, subjective probability, human judgment, psychological methods

Most psychological research on Bayesian reasoning since the 1970s has used a type of problem that tests a certain kind of statistical reasoning performance. The subject is given statistical facts within a hypothetical scenario. Those facts include a base-rate statistic and one or two diagnostic probabilities. The subject is meant to use that information to arrive at a “posterior” probability estimate. For instance, in one well-known problem (Eddy, 1982) the subject encounters the following:

The probability of breast cancer is 1% for a woman at age forty who participates in routine screening. If a woman has breast cancer, the probability is 80% that she will get a positive mammography. If a woman does not have breast cancer, the probability is 9.6% that she will also get a positive mammography. A woman in this age group had a positive mammography in a routine screening. What is the probability that she actually has breast cancer? __ %.

The information in such problems can be mapped onto common expressions that use *H* as the focal hypothesis, ¬*H* as the mutually-exclusive hypothesis, and *D* as datum: *P*(*H*), the prior (often equated with the base-rate) probability of the hypothesis; *P*(*D*|*H*), the true-positive rate; and *P*(*D*|¬*H*), the false-positive rate. In the mammography problem, *P(H)* = 0.01, *P*(*D*|*H*) = 0.80, and *P*(*D*|¬*H*) = 0.096. Furthermore, *P*(¬*H*) = 1 – *P*(*H*) = 0.99. The estimate queried is *P*(*H*|*D*).

Bayes' theorem states:

$$P(H|D)=\frac{P(H)P(D|H)}{P(H)P(D|H)+P(\neg H)P(D|\neg H)}.$$

Thus, it yields a posterior probability of 0.078 in the mammography problem. Yet even the majority of physicians who were queried by Eddy (1982) gave estimates roughly one order of magnitude higher (i.e., 0.70–0.80).

Well-established findings such as these have supported the view that expert and naïve subjects alike are non-Bayesian (Kahneman and Tversky, 1972). A common explanation is that people neglect base-rate information, which is not tracked by the intuitive heuristics they use to reach an estimate (Kahneman and Tversky, 1972, 1973). For instance, if base rates were neglected in the mammography problem,

$$P\left(H|D\right)=\frac{0.80}{0.80+0.096}\approx 0.89.$$

This estimate is closer to the modal estimate but is still off by about ten percentage points. Another explanation is that people commit the *inverse fallacy*, confusing *P*(*H*|*D*), which they are asked to estimate, with *P*(*D*|*H*), which is provided (Koehler, 1996). In the mammography problem, this explanation fits the data well because *P*(*D*|*H*) = 0.80. The inverse fallacy can also explain patterns of deviation from Bayes' theorem in tasks that hold constant base rates for alternative hypotheses (Villejoubert and Mandel, 2002).

It is also known that steps can be taken to increase agreement with Bayes' theorem. Since Bayes' theorem can be simplified as

$$P\left(H|D\right)=\frac{f\left(D\cap H\right)}{f\left(D\right)},$$

task reformulations that directly provide these values or make them easily computable increase the proportion of Bayesian responses (e.g., Gigerenzer and Hoffrage, 1995; Hoffrage et al., 2002; Ayal and Beyth-Marom, 2014). Such formulations of evidence reduce computational steps and may also effectively trigger awareness of the correct solution, much as eliciting logically-related probability estimates (e.g., of binary complements) in close proximity rather than far apart improves adherence to the additivity property (Mandel, 2005; Karvetski et al., 2013). Natural frequency representations, which reveal nested-set relations among a reference class or representative sample (Gigerenzer and Hoffrage, 1995; Cosmides and Tooby, 1996), lend themselves easily to such simplification and have been shown to improve Bayesian reasoning. For instance, Bayesian responses to the mammography problem more than doubled when it was presented in natural-frequency format (Gigerenzer and Hoffrage, 1995). Although the theoretical bases of such improvements are debated (e.g., Barbey and Sloman, 2007, and continuing commentaries), most agree that substantial improvement in conformity to Bayes' theorem is achievable in this manner.

Bayesian reasoning also benefits from the use of visual representations of pertinent statistical information, such as Euler circles (Sloman et al., 2003) and frequency grids or trees (Sedlmeier and Gigerenzer, 2001), which further clarify nested-set relations. For instance, Figure 1 shows how the natural-frequency version of the mammography problem could be represented with a frequency tree to help individuals visualize the nested-set relations and how such information ought to be used to compute the posterior probability.

**Figure 1:**
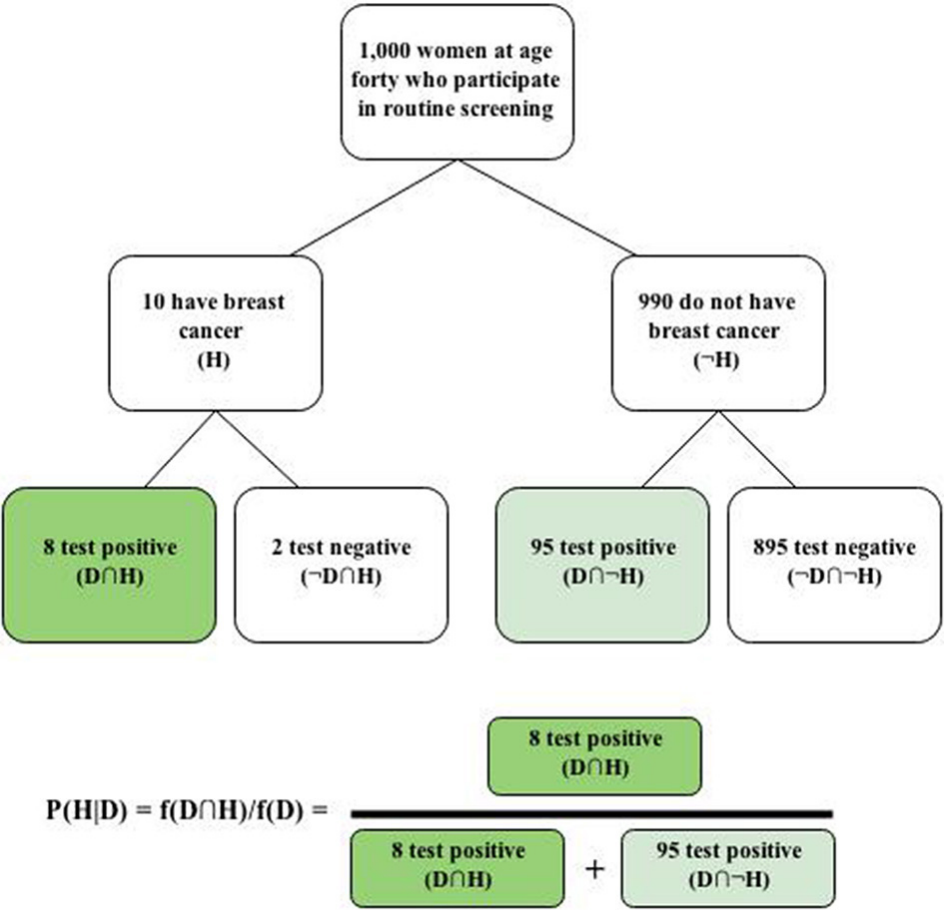
**Frequency tree and solution for the mammography problem**.

## Observations

A remarkable feature of the standard approach to studying Bayesian reasoning is its inability to reveal how people *revise* their beliefs or subjective probabilities in light of newly acquired evidence. That is, in tasks such as the mammography problem, information acquisition is not staged across time (real or hypothetical), and researchers typically do not collect multiple “prior” and “posterior” (i.e., revised) probability assessments.

It is instead conveniently assumed that the base rate represents the subject's prior belief, *P*(*H*), which the subject updates in light of “new” evidence, *D*. It is somewhat ironic that advocates of base-rate neglect have not noted (let alone warned) that, if people ignore base rates, it may be unwise to assume they represent the subject's prior probability. Would that not imply that the subject ignores his or her own prior probability?

Priors need not equal base rates, as many have noted (e.g., de Finetti, 1964; Niiniluoto, 1981; Levi, 1983; Cosmides and Tooby, 1996). The prior, *P*(*H*), is in fact a *conditional* probability corresponding to one's personal probability of *H*, given all that they know prior to learning *D* (Edwards et al., 1963; de Finetti, 1972). In all real-life cases where no single, relevant base rate is ever explicitly provided, people may experience considerable uncertainty and difficulty in deciding precisely which base rate is the most relevant one to consider. For instance, imagine that the test result in the mammography problem is for a specific, real woman and not just an abstract one lacking in other characteristics. If her prior for *H* is contingent on the presence or absence of some of those characteristics, one could see how the base rate provided in the problem might be more or less relevant to the woman's particular case. If she has several characteristics known to elevate a woman's risk of breast cancer, then simply using the base rate for 40-year-old women as her prior would bias her revised assessment by leading her to underestimate the risk she faces. Conversely, she may have a configuration of characteristics that make her less likely than the average 40-year-old woman to develop breast cancer, in which case using the base rate as her prior would cause her to overestimate objective risk.

Clearly, the ideal base rate in such personal cases would be a sample of people who are just like the patient, yet since each of us is unique no such sample exists. In the absence of a single, ideal base rate, one must decide among a range of imperfect ones—a task involving decision under uncertainty. It might be sensible for the woman getting the screening to anchor on a relevant, available base rate, such as for women in her cohort, and then adjust it in light of other diagnostic characteristics that she knows she possesses. Yet, if people are overly optimistic (Taylor and Brown, 1988; Weinstein, 1989), we might anticipate systematic biases in adjustment, with underweighting of predisposing factors and overweighting of mitigating factors. This point about the possible role of motivated cognition also brings a key tenet of subjective Bayesianism to the fore—namely, that different individuals with access to the same information could have different degrees of belief in a given hypothesis, and they may be equally good Bayesians as long as they are equally respectful of static and dynamic coherence requirements (Baratgin and Politzer, 2006).

Given that standard Bayesian reasoning tasks involve no assessment of a prior probability, they should be seen for what they are: conditional probability judgment tasks that require the combination of statistical information. When that information is fleshed out, it reveals the fours cells of a 2 × 2 contingency table, where *a = f (H ∩ D)*, *b = f (H ∩ ¬ D)*, *c = f (¬ H∩ D)*, and *d = f (¬H ∩ ¬D)*. Going from left to right, the four boxes in the lowest level of the frequency tree in Figure 1 correspond to cells *a–d*, which have received much attention in the causal induction literature (Mandel and Lehman, 1998). We can restate Bayes' theorem as the following cell-frequency equalities, corresponding to short and long expressions given earlier, respectively:

$$\begin{array}{l} P\left(H|D\right)=\frac{a}{a+c}\\ \;=\frac{\begin{array}{c} \left(a+b\right)/\left(a+b+c+d\right)\times \\ a/\text{​}\left(a+b\right) \end{array}}{\begin{array}{c} \left(a+b\right)/\left(a+b+c+d\right)\times \\ a/\text{​}\left(a+b\right)+\left(c+d\right)/\left(a+b+c+d\right)\\ \times c/\left(c+d\right) \end{array}}. \end{array}$$

From this perspective, it is perhaps unsurprising why a greater proportion of subjects conform to Bayes theorem when they are given the frequencies *a–d* than when they are instead given the values equal to (*a* + *b*)/(*a* + *b* + *c* + *d*), *a/(a* + *b*), and *c*/(*c* + *d*). That is, frequencies *a*–*c* support the easy computation of *a*/(*a* + *c*). However, those improvements in performance, which pertain to static coherence constraints (Baratgin and Politzer, 2006), do not speak to other important facets of Bayesian reasoning, such adherence to dynamic coherence constraints, which are fundamental to Bayesian belief revision (Seidenfeld, 1979).

I do not intend for my observations to imply that the well-established findings I summarized earlier are incorrect. However, I believe greater care should be taken in labeling the type of performance measured in such experiments. “Statistical inference” would seem to be more appropriate than “Bayesian reasoning” given the limitations I have noted.

Future research on Bayesian reasoning would benefit from a richer conceptualization of what it is to “be Bayesian” and from better discussion of whether being non-Bayesian is necessarily irrational (Lewis, 1976; Walliser and Zwirn, 2002; Baratgin and Politzer, 2006). Future work would also benefit by breaking free of the typical methodological approach exemplified by the mammography problem. One avenue would be to collect prior and posterior assessments from subjects in experiments where information acquisition is staged (e.g., Girotto and Gonzalez, 2008), or where temporal staging is at least an important characteristic of the described problem, such as in the Monty Hall problem (Krauss and Wang, 2003) and Sleeping Beauty problem (Elga, 2000; Lewis, 2001). Another promising line involves assessing people's prior distributions for different types of real events (e.g., Griffiths and Tennenbaum, 2006).

The staging of information with repeated assessments was in fact a common methodological approach in Bayesian research prior to the 1970s, culminating in the classic work on conservatism by Ward Edwards and others (for a review, see Slovic and Lichtenstein, 1971). Such approaches could be revisited in new forms and contrasted with other methods of information staging, such as the trial-by-trial information acquisition designs used in causal induction (e.g., Kao and Wasserman, 1993; Mandel and Vartanian, 2009) or category learning (e.g., Gluck and Bower, 1988; Shanks, 1990) studies.

For example, Williams and Mandel (2007) presented subjects with 28 problems prompting them for a conditional probability judgment. In each problem, subjects first saw 20 patient results presented serially. The subject saw whether the patient carried a virus hypothesized to cause a particular illness and whether the patient had the illness or not. Sample characteristics were varied so that *P*(*H*|*D*) ranged from 0 to 1 over seven probability levels across the problems. Subjects exhibited a form of conservatism (cf. Edwards, 1968), overestimating low probabilities and underestimating high probabilities. The task illustrates the value of breaking free of the standard problem set. First, the trial-by-trial design better represents the information acquisition environment that ecological rationality theorists (e.g., Gigerenzer and Hoffrage, 1995; Cosmides and Tooby, 1996), have described as natural. That is, information acquisition in that task is more natural than in natural-frequency versions of standard problems because no statistical information is presented to the subject in written form. Rather, subjects learn about each case serially, more like they would have in the Paleolithic Era. Second, the design gets researchers away from studying average responses to a single problem with a unique data configuration. The authors would not have been able to detect conservatism if they had not explored problems for which the mathematical probabilities subjects were asked to judge covered the full probability range. Third, the induction paradigm, which presents information on cells *a–d* to subjects, easily lends itself to studying subjective cell importance, which can help take the cognitive processes subjects use to arrive at their judgments out of the proverbial black box. For instance, Williams and Mandel (2007) found that, when asked to assign subjective importance ratings to each of the fours cells, subjects assigned weight to irrelevant information, such as focusing on *¬D* cases when asked to judge P(*H*|*D*), causing an underweighting of relevant information.

The issues I have raised, non-exhaustive as they are, draw attention to some important problems with the conventional approach to studying Bayesian reasoning in psychology that has been dominant since the 1970s. Rather than fostering pessimism, I hope my comments illustrate that there are good opportunities for future work to advance our understanding of how people revise or update their beliefs.

### Conflict of interest statement

The author declares that the research was conducted in the absence of any commercial or financial relationships that could be construed as a potential conflict of interest.
